# Detecting Thermal Cloaks via Transient Effects

**DOI:** 10.1038/srep32915

**Published:** 2016-09-08

**Authors:** Sophia R. Sklan, Xue Bai, Baowen Li, Xiang Zhang

**Affiliations:** 1 Department of Physics, Massachusetts Institute of Technology, Cambridge, Massachusetts 02139, USA; 2 Department of Mechanical Engineering, University of California, Berkeley, California 94720, USA; 3 Department of Mechanical Engineering, University of Colorado Boulder, Colorado 80309, USA; 4 Department of Electrical and Computer Engineering, National University of Singapore, 4 Engineering Drive 3, Singapore 117583, Republic of Singapore; 5 Department of Physics and Centre for Computational Science and Engineering, National University of Singapore, Singapore 117546, Republic of Singapore; 6 NUS Graduate School for Integrative Sciences and Engineering, National University of Singapore, Kent Ridge 119620, Republic of Singapore; 7 NSF Nanoscale Science and Engineering Centre, 3112 Etcheverry Hall, University of California, Berkeley, California 94720, USA; 8 Materials Sciences Division, Lawrence Berkeley National Laboratory, 1 Cyclotron Road, Berkeley, California 94720, USA

## Abstract

Recent research on the development of a thermal cloak has concentrated on engineering an inhomogeneous thermal conductivity and an approximate, homogeneous volumetric heat capacity. While the perfect cloak of inhomogeneous *κ* and inhomogeneous *ρc*_*p*_ is known to be exact (no signals scattering and only mean values penetrating to the cloak’s interior), the sensitivity of diffusive cloaks to defects and approximations has not been analyzed. We analytically demonstrate that these approximate cloaks are detectable. Although they work as perfect cloaks in the steady-state, their transient (time-dependent) response is imperfect and a small amount of heat is scattered. This is sufficient to determine the presence of a cloak and any heat source it contains, but the material composition hidden within the cloak is not detectable in practice. To demonstrate the feasibility of this technique, we constructed a cloak with similar approximation and directly detected its presence using these transient temperature deviations outside the cloak. Due to limitations in the range of experimentally accessible volumetric specific heats, our detection scheme should allow us to find any realizable cloak, assuming a sufficiently large temperature difference.

The ability to render an object invisible has been a goal since the days of mythology and the Ring of Gyges. It is only recently that invisibility became a plausible subject of inquiry thanks to theoretical advances in electromagnetism^1^,^2^,^3^,^4^,^5^,^6^. Such cloaks fulfilled the two basic elements of invisibility: anything hidden inside was isolated as if hidden by a perfect insulator (mean values still transmittable) and the perfect insulator had no scattering. This first requirement typically entailed singular, anisotropic materials, while the second required inhomogeneity. These extreme material requirements turned attention to reduced cloaks which merely approximate perfect cloaking^7^,^8^,^9^,^10^ or conditions where these constraints are relaxed^11^,^12^,^13^,^14^,^15^,^16^,^17^. This in turn led to the study the detectability of these cloaks^18^,^19^,^20^,^21^,^22^. Concurrently, cloaking was extended to other classes of electromagnetic phenomena^23^,^24^,^25^,^26^,^27^,^28^,^29^, wave equations^30^,^31^,^32^,^33^,^34^,^35^,^36^,^37^. and diffusion equations^38^,^39^,^40^,^41^,^42^,^43^,^44^,^45^,^46^,^47^,^48^,^49^,^50^,^51^ (refs 52, 53 provide a review of these last categories).

The diffusive cloaks found greatest success with the heat equation:


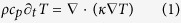


(where *ρ* is the density, *c*_*p*_ the specific heat capacity, *T* temperature, and *κ* the thermal conductivity), so we shall confine our attention to thermal cloaks and then generalize our results to other diffusion effects. Because the steady-state temperature is independent of the volumetric heat capacity *ρc*_*p*_, cloaking has focused on engineering *κ* with *ρc*_*p*_ constant. This has led to the widespread acceptance of three related classes of diffusive cloaks. First, there are strictly steady-state cloaks that ignored *ρc*_*p*_ through the assumption that they would only be used in a steady state. While the utility of such a device is limited (truly steady-state conditions are unlikely to be maintained outside of specialized circumstances), they unquestionably work as expected. Their success, however, led to a second class of cloaks, which sought to extend these steady-state designs to time-varying and transient temperatures. For example Schittny *et al*.^43^ adopt a theoretical approach from ref. 42 (which assumed constant *ρc*_*p*_ as an explicit approximation) while still claiming to achieve transient thermal cloaking. Indeed, they even found experimentally that their specific heat capacity varied between their materials but sought to correct for it to maintain a homogeneous *ρc*_*p*_. In addition, Ma *et al*.^45^, simplify their design by assuming that *ρc*_*p*_ commutes with spatial derivates, i.e. that homogeneity of *ρc*_*p*_ is obtained to good approximation. The third class of diffusive cloaks were chemical diffusion cloaks^49^,^50^, which are governed by ∂_*t*_*n* = ∇ · (*D*_*n*_∇*n*) for chemical concentration *n* and diffusion constant *D*_*n*_. There, researchers again assumed that *ρc*_*p*_ was negligible based upon the performance of steady-state thermal cloaks, and therefore that steady-state thermal cloak designs could be extended to transient effects in chemical diffusion (even though this analogy maps *ρc*_*p*_ to unity everywhere). Thus, although the steady-state cloak was developed for the limited frequency range of *ω* = 0, there now exists a broad class of cloaks (which we refer to collectively as steady-state cloaks due to their common origin) which assume that careful engineering of *κ* is sufficient to create a perfect cloak in any regime. Note that an alternative approach to thermal cloaking was also presented in ref. 54, which used scattering cancellation theory. The theoretical justification for this type of cloaking is thus outside of the analysis we develop here, although it is worth noting that they explicitly engineer *ρc*_*p*_ and *κ*. However, as a complementary media method, it only cloaks specific objects (or at most a class of objects all having the same effective parameters), whereas transformation media cloaks work for arbitrary objects. In addition, the technique works by cancelling out finite terms in the multipole expansion of the cloaked object’s scattering cross section, and so remains detectable using higher-order terms in the expansion (e.g. the quadrupole term in their example).

In this paper, we show a homogeneous *ρc*_*p*_ results in a detectable, transient signal. Under changing boundary conditions, a thermal cloak will flicker and become visible, although it will help to obscure anything hidden inside it. The implications of this imperfection can be seen by considering a faulty cloak with one observer hidden inside and another searching outside (see Fig. 1). The searcher can send out signals and detect the diffuse “scattering” that is reflected back. Furthermore, they can search for signals emanating from the cloak’s interior − thereby determining the material composition or temperature distribution hidden inside. Conversely, the observer hiding in the cloak can detect incoming signals to observe any searchers and eavesdrop on the outside. Moreover, by sending out their own signals and detecting them, they can confirm that the cloak is present and functioning.

## Results

**Figure 1:**
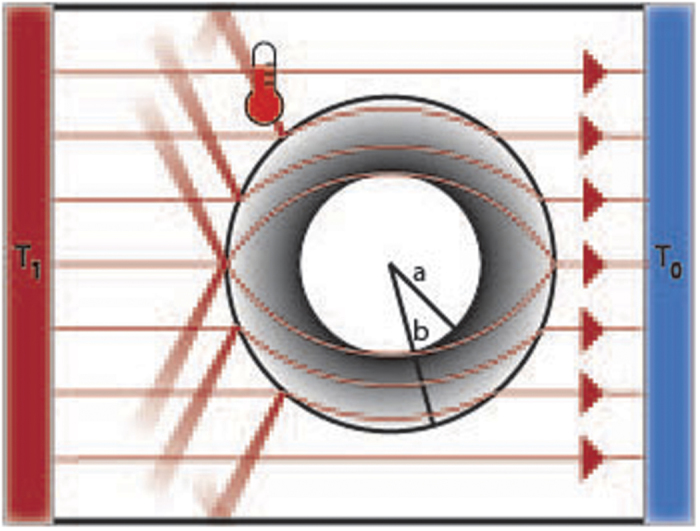
Simple model for detecting a cloak. Cloaked region of radius *a* inside a cloak of radius *b* is protected from outside searchers. To detect the cloak, it is surrounded by two heat baths (red and blue rectangles) in a thermally isolated domain. Heat (red lines) flows through the cloak and emerges without distortion. However, for an imperfect cloak heat is also scattered. The scattered heat diffuses, but a thermometer placed near the cloak can detect it.

We begin by considering the analytic solution to eq. 1 for the cylindrical perfect cloak (PC) (assuming no *z* dependence). For a homogeneous medium (*κ* = *κ*_0_, *ρc*_*p*_ = *ρ*_0_*c*_*p*0_), source-free medium the solution can be expressed as a linear combination of the fundamental solutions


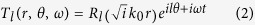


where *l* the rotational symmetry eigenvalue, *R*_*l*_ is a modified Bessel function of the first or second kind (*I*_*l*_ and *K*_*l*_ respectively), 
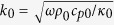
, and *ω* is a frequency >0 (the steady state of *ω* = 0 is discussed in the supplement). A PC of interior radius *a* and exterior radius *b* (Fig. 1) is constructed from


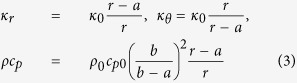


the solution becomes


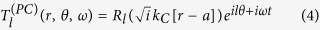


where *k*_*C*_/*k*_0_ = *b*/(*b* − *a*). Whereas, for a steady-state cloak (SSC) *κ* is the same as eq. 3 but *ρc*_*p*_ = (*b*/(*b* − *a*))*ηρ*_0_*c*_*p*0_ (*η* a mismatch parameter, *η* = 1 is eq. 3 evaluated at *r* = *b*), the lowest order perturbation is


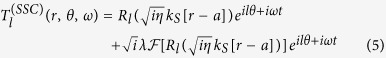


where 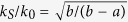, 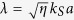 and 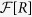 is given in the supplement (along with the full analytic solution). Crucially, *λ* determines the strength of the perturbation, meaning the effectiveness of an impedance matched SSC (*η* = 1) is proportional to the size of the cloaked region over the diffusion length. For reference, the various cloaks considered in this paper are summarized in Table 1.

**Figure 2:**
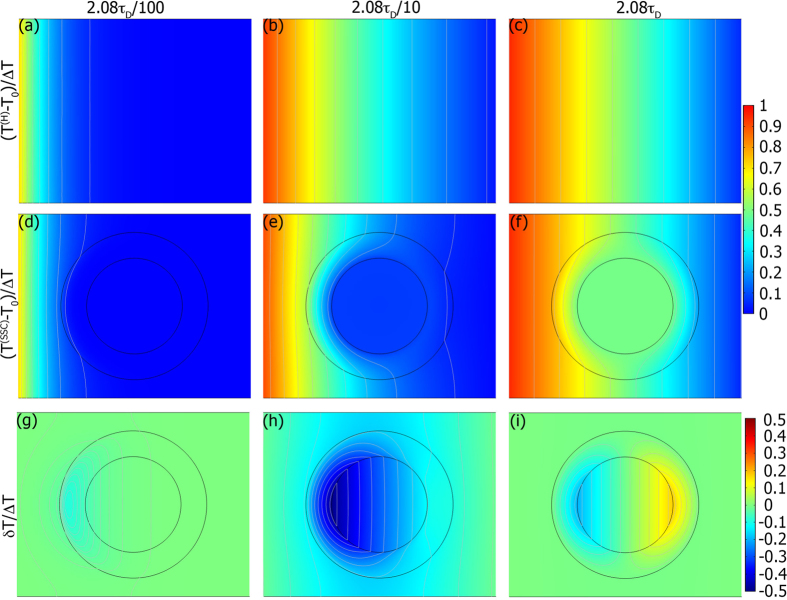
Simulated temperature snapshots for mismatched SSC (*η* = *b*/(*b* − *a*)). Columns correspond to 2.08*τ*_*D*_/100, 2.08*τ*_*D*_/10, and 2.08*τ*_*D*_ respectively. Rows correspond to the homogeneous case (no cloak), SSC, and *T*^(*SSC*)^ − *T*^(*H*)^. Black circles denote the location of the cloak (for reference in the homogeneous case), colored domains are isotherms, and grey lines are constant separation isotherms.

Since the solution to eq. 5 is not a tabulated function, we use COMSOL multiphysics^55^ to solve eq. 1 directly for the SSC. Following the most common test of a cloak, we model the cloak in a rectangular region where one pair of ends are held at fixed temperature and the other pair admit no heat (Fig. 1). Given the linearity of eq. 1, one boundary is set to 0 (as is the initial *T*) and the other to 1 (Δ*T* ≡ 1). It is helpful to use the natural units of *L* (the separation of the heat sources) and the diffusion time *τ*_*D*_ = *L*^2^*ρ*_0_*c*_*p*0_/*κ*_0_ (all parameters values are in the supplement). Figure 2 is the result of these calculations. Each column is a snapshot at a different time. The first row is the homogeneous background that would be observed if there was no cloak, the second is the solution to SSC (with *η* = *b*/(*b* − *a*) to increase contrast), and the third is the difference 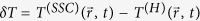. This deviation *δT* is what must be detected to reveal a cloak. Initially *δT* is small and mostly localized to where the cloak has been heated (see Fig. 2g). Later (Fig. 2h) *δT* grows and is clearly observed outside the cloak. Finally in the steady state (Fig. 2i) invisibility is restored, as expected for a SSC (*δT* ≠ 0 confined to within the cloak).

**Figure 3:**
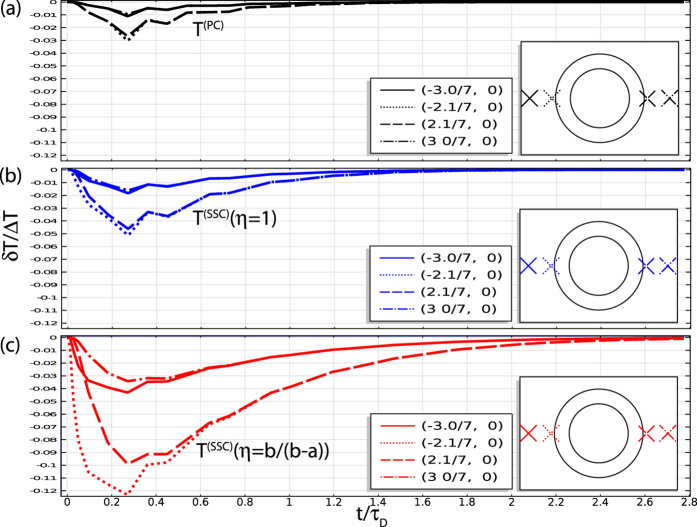
Temperature deviation *δT*/Δ*T* for representative points outside the cloak as a function of time. Black (**a**), blue (**b**), and red (**c**) curves correspond to the PC, impedance matched SSC, and impedance mismatched SSC. Line styles correspond to individual points, as shown in the inset.

To clarify the time-dependence of *δT* we select several points outside the cloak and compare *δT* for SSC with *η* = 1 (i.e. impedance matched, cloak has the same properties as a PC at *r* = *b*), *η* = *b*/(*b* − *a*) >1 (impedance mismatch but 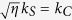), and the PC in Fig. 3. As we prove in the supplement, *δT* = 0 outside the cloak for the PC (i.e. that a perfect cloak is perfect), so the non-zero *δT* must be a numerical artifact of discretizing *κ* and thereby removing *κ*_*θ*_ → ∞ (removal of this singularity introduces scattering fields which scale to lowest order like 1/ln *k*_*C*_*δ*, where *δ* is the deviation at the inner boundary. This is further corroborated by the penetration of heat into the cloaked region). However, for both SSC models |*δT*^(*SSC*)^| > |*δT*^(*PC*)^| outside the cloak. This is the effect a homogeneous *ρc*_*p*_. Note that the position dependence of *δT* is approximately just a scaling factor so that the peaks all nearly coincide instead of being separated by a propagation time. Both this and the time dependence (linear growth for small *t*, exponential decay for large) are derived in the supplement, where we show that this separable space dependence implies that *δT* is dominated by a small number of Fourier-modes.

The results of Fig. 3a present a finite element, (i.e. discretized) approximation of the exact inhomogeneity relations that constitute a PC. They are therefore not just a numerical approximation of an analytic cloak but also a computational approximation of how a near ideal, experimentally realizable cloak might behave. The reduction in the cloak’s scattering that we gain by incorporating the inhomogeneity of *ρc*_*p*_ is reflected drop in the temperature deviation peaks between Fig. 3a,b (a factor of 2 for the impedance matched SSC and a factor of 4 for the impedance mismatched SSC).

**Figure 4:**
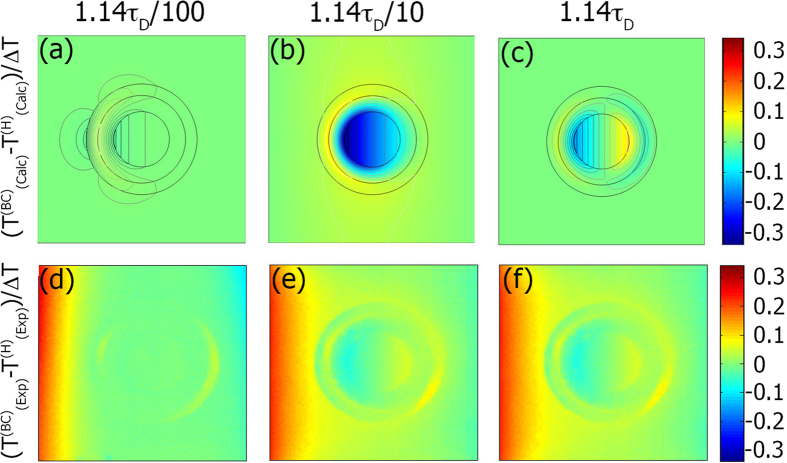
Comparison of simulations and experimental for the BC. Columns correspond to 1.14*τ*_*D*_/100, 1.14*τ*_*D*_/10, and 1.14*τ*_*D*_ respectively. Rows correspond to *δT* for the simulation and experiment respectively.

While the SSC model uses a realistic *ρc*_*p*_, it still contains an idealized *κ*. The exact inhomogeneity and anisotropy profiles of eq. 3 are not physically realizable. Thus, experimental verification of our predictions for the SSC is impeded by its use of an idealized *κ*. In real thermal cloaks, rings of discretized, constant *κ* are used in an approximation of this ideal inhomogeneity curve. Hence, for a direct comparison, we move from the SSC model that we have developed to the case of a bilayer cloak (BC)^48^ (simulations and further experimental data for the BC are in the supplement). The BC is particularly interesting to consider as it is a SSC that was derived directly from Laplace’s equation rather than a coordinate transformation (similar to^54^). In Fig. 4 we plot the normalized temperature deviation for the simulated BC and our experimental realization. This shows a good agreement, with a slight discrepancy near the boundaries of the system. This is due to a slight difference in the experimental temperature gradients applied to the BC and homogeneous cases.

**Figure 5:**
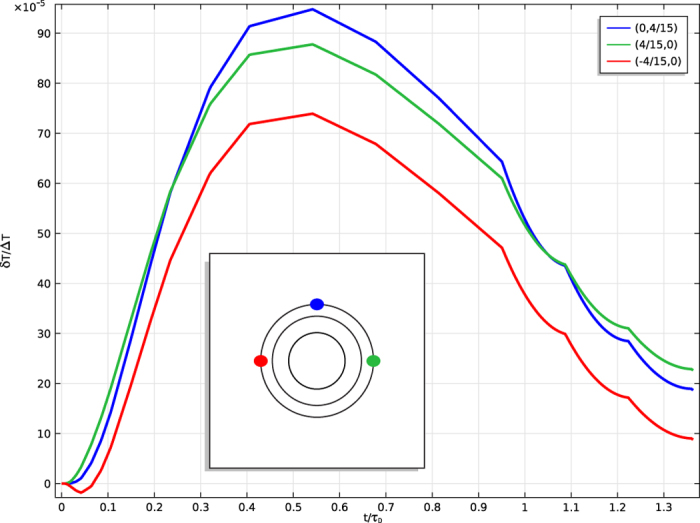
Deviation of temperature profiles with changing cloaked object composition (*T*^(*cloak*+*object*)^ − *T*^(*cloak*)^)/Δ*T* for representative points outside the cloak as a function of time. Color corresponds to different points (see inset for key).

Finally, we turn to the question of detecting objects hidden inside a cloak. For the PC and the SSC 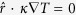 at the boundary (*κ*_*r*_ = 0), so there should be no heat transferred and therefore no discernable signal (although, as in ref. 18, this is extremely sensitive to deviations of *κ*_*r*_ from 0 and as in refs 23, 24 and 37 even a PC will transmit the mean value of *T* at the boundary). However, taking the BC and changing the material hidden inside will effect the temperature distribution. An exterior temperature profile like those considered above must pass through the cloak twice (entering and exiting), so the cloak’s ability to suppress detection is stronger here than in the case of hiding the cloak. In particular, simulations of the BC with different materials inside differ by less than 0.1% (see Fig. 5). Assuming a thermometer of sensitivity of 0.2 K, a gradient of over 200 K would be necessary for the determination of the material hidden within the cloak (whereas merely detecting the presence of the BC requires a temperature difference of 3.64 K).

**Figure 6:**
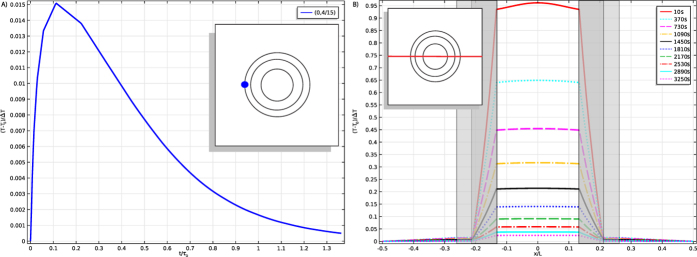
Temperature response of the BC used as an insulator. Plot (**a**) shows the time dependence of a representative point outside the cloak while plot (**b**) shows the spatial dependence of a slice through the cloak (see inset) at various times (in seconds). The fluctuation near the inner boundary is a numerical artifact of the discontinuity in initial temperature.

On the other hand, one could try to detect the temperature distribution hidden by the cloak, rather than the material. In this case, heat initially confined to the cloak would diffuse out and only pass through the cloak once. Simulations in this case show a detectable signal of 1.5% (see Fig. 6, computational details in the supplement), meaning that a thermometer of 0.2 K sensitivity could by used to detect a temperature difference of at least 13.3 K from the background. It is therefore likely that heat sources can be detected through the cloak, i.e. that it acts like an imperfect insulator. Comparing the efficacy of the BC as an insulator to that of a thermal insulator with properties equal to the insulating layer of the BC (essentially, removing one of the layers) indicates that the BC is no better at suppressing this diffusion of heat into the environment. This suggests that realizable cloaks (i.e. those without a perfectly insulating inner boundary) are no better than conventional insulators for maintaining a temperature difference. This is not entirely surprising if we think of the thermal cloak as a composite or inhomogeneous wall, but it reveals a way in which thermal cloaks deviate for our intuition of what cloaking means (indeed, suggestions that thermal cloaks can hide hot spots or the thermal signatures of objects are somewhat commonplace in the community^54^, is one such example). Moreover, even a PC^23^,^24^ with a perfectly insulating inner boundary would still not prevent the matching of the average temperature inside and outside of the cloak, so this set up should still eventually equilibrate. For the PC, though, this should take much longer and be a much smaller signal, so the lack of a perfect insulating layer aids experimental detectability of the internal temperature distribution.

## Discussion

We have shown that a SSC can be detected by its transient response. Because the distinction between a PC and a SSC is just *ρc*_*p*_, the ability to engineer the volumetric heat capacity is necessary to prevent the *ω* ≠ 0 response from revealing the cloak. However, the narrow range of *ρc*_*p*_ in currently available materials makes it extremely difficult to design this inhomogeneity (indeed, even efforts to construct a “transient” thermal cloak have assumed constant *ρc*_*p*_^43^,^45^). This is particularly true for other classes of diffusion cloaks where the analog of *ρc*_*p*_ is necessarily constant everywhere^49^,^50^. It remains an open question, however, if a diffusive cloak (thermal or otherwise) could be designed to make its time-dependent response undetectable in practice even if the response exists in principle.

## Methods

### Analytic solutions

Given the heat equation with homogeneous materials


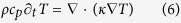


in polar coordinates we take the Fourier transform of time and use a separable solution *T*(*r, θ, t*) = *R*(*r*)*e*^*ilθ*^*e*^*iωt*^ giving


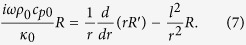


This is the differential equation for a modified Bessel function (*I*_*l*_(*z*) or *K*_*l*_(*z*)) of 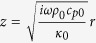 for *ω* ≠ 0^56^. The time-dependent solution is therefore


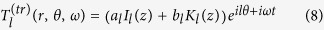


For the steady state of *ω* = 0 the solutions become the solution to Laplace’s equation


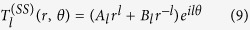


for *l* ≠ 0 and


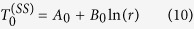


for *l* = 0. The general solution is therefore 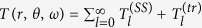.

For a perfect cloak


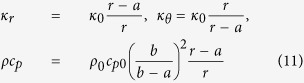


we can make the coordinate transformation


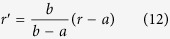


to reduce the solution in the primed coordinates to the homogeneous case.

For a steady-state cloak (*κ* as for the perfect cloak, *ρc*_*p*_ = *ρ*_0_*c*_*p*0_(*b*/(*b* − *a*))*η*, i.e. evaluating *ρc*_*p*_ at *r* = *b* when *η* = 1) no transformation will reproduce a homogeneous solution. Using 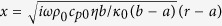 and separation of variables we find


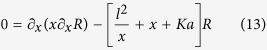


where 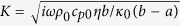. This can be solved by the method of Frobenius 
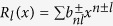
 with recurrence relation


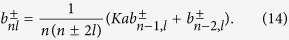


This relation is exact, but additional insight can be gained by expanding the solution by powers of *Ka*. For even terms in the series this is


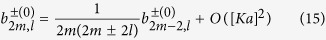


which is the same as series expansion for *I*_*l*_ and *K*_*l*_ respectively. On the other hand, for odd terms it becomes


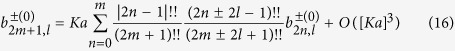


Because 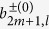 is completely determined by 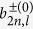 the odd terms are therefore a function of the modified Bessel functions. Ergo, we term these components 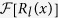. A similar derivation can be carried out for a spherical cloak where *l* becomes half-integer instead of integer.

### Computational and Experimental Methods

For the PC and SSC we use COMSOL multiphysics to model a rectangular domain of dimensions *L* = 70 mm by *L*_⊥_ = 50 mm centered around a cloak of dimension *a* = 13 mm, *b* = 20 mm. The background medium is *κ*_0_ = 71.4 W/m · K, *ρ*_0_ = 2100 kg/m^3^, and *c*_*p*0_ = 1000 J/kg · K. This gives a diffusivity of *D* = *κ*_0_/*ρ*_0_*c*_*p*0_ = 3.4 · 10^−5^ m^2^/s and diffusion timescale *τ*_*D*_ = *L*^2^/*D* = 144.12 s. The initial temperature was 293.15 K with thermal baths at 300 K, and *T*_0_ = 293.15 K giving a Δ*T* of 6.85 K. After confirming that the simulations were invariant under a change of scale we use the natural units of *x*/*L, y*/*L, t*/*τ*_*D*_, (*T* − *T*_0_)/Δ*T*.

For the BC, we follow^48^ and model it as rectangular domain of dimensions *L* = 45 mm by *L*_⊥_ = 45 mm centered around a cloak with hidden region of size *a* = 6 mm, first layer of *r*_2_ = 9.5 mm, and second layer of *b* = 12 mm. The background medium is *κ*_0_ = 2.3 W/m · K, *ρ*_0_ = 2000 kg/m^3^, and *c*_*p*0_ = 1500 J/kg · K, the outer layer’s medium is *κ*_1_ = 9.8 W/m · K, *ρ*_1_ = 8440 kg/m^3^, and *c*_*p*1_ = 400 J/kg · K, the inner layer’s medium is *κ*_2_ = 0.03 W/m · K, *ρ*_2_ = 50 kg/m^3^, and *c*_*p*2_ = 1300 J/kg · K, and the interior medium is *κ*_3_ = 205 W/m · K, *ρ*_3_ = 2700 kg/m^3^, and *c*_*p*3_ = 900 J/kg · K. This gives a diffusivity of *D*_0_ = *κ*_0_/*ρ*_0_*c*_*p*0_ = 7.67 · 10^−7^ m^2^/s and diffusion timescale 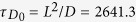 s. The initial temperature was 273.15 K with thermal baths at 333.15 K, and *T*_0_ = 273.15 K giving a Δ*T* of 60 K. The experimental tests of the BC were performed with the same setup, following the procedure outlined in ref. 48.

As for the ability of a cloak to insulate a cloaked object and thus disguise the temperature profile, it is helpful to use different boundary and initial conditions. Instead of applying a thermal gradient across the boundaries, the cloaked region is initially set to 60 K above the background (and cloak) at 273.15 K (These values are then rescaled to 1 and 0). Because the thermal baths are at fixed temperature and perfectly absorb heat flux, energy is not conserved in this simulation and so the steady state should have all the heat removed from the cloak.

## Additional Information

**How to cite this article**: Sklan, S. R. *et al*. Detecting Thermal Cloaks via Transient Effects. *Sci. Rep.* **6**, 32915; doi: 10.1038/srep32915 (2016).

## Supplementary Material

Supplementary Information

## Figures and Tables

**Table 1:** Equations for cloaks considered in this paper.

Cloak	*κ*_*r*_/*κ*_0_	*κ*_*θ*_/*κ*_0_	*ρc*_*p*_/*ρ*_0_*c*_*p*0_
PC	(*r* − *a*)/*r*	*r*/(*r* − *a*)	[*b*/(*b* − *a*)]^2^(*r* − *a*)/*r*
SSC (M)	(*r* − *a*)/*r*	*r*/(*r* − *a*)	*b*/(*b* − *a*)
SSC (Mis)	(*r* − *a*)/*r*	*r*/(*r* − *a*)	[*b*/(*b* − *a*)]^2^
BC {*r* ∈ (*a, r*_1_)}	*κ*_1_/*κ*_0_	*κ*_1_/*κ*_0_	*ρ*_1_*c*_*p*1_/*ρ*_0_*c*_*p*0_
BC {*r* ∈ (*r*_1_, *b*)}	*κ*_2_/*κ*_0_	*κ*_2_/*κ*_0_	*ρ*_2_*c*_*p*2_/*ρ*_0_*c*_*p*0_

Perfect cloak (PC), impedance matched steady-state cloak (SSC (M)), impedance mismatched SSC (SSC (Mis)) (*η* ≡ *b*/(*b* − *a*)), and bilayer cloak (BC). Inner layer of the cloak has radius *a*, outer layer radius *b*, as in Fig. 1.
