# Supplementary material for: Detecting Thermal Cloaks via Transient Effects
Source: Sci Rep. 2016 Sep 8;6:32915. doi: 10.1038/srep32915 (PMC5015050; doi:10.1038/srep32915)
Supplement: Supplementary Information [file srep32915-s1.pdf]

Detecting Thermal Cloaks via Transient Effects
Supplementary Materials

Sophia R. Sklan,[1,2,3] Xue Bai,[4,5,6] Baowen Li*,[2,3] and Xiang Zhang[2,7,8]

[1]Department of Physics, Massachusetts Institute of Technology, Cambridge, Massachusetts 02139, USA
[2]Department of Mechanical Engineering, University of California, Berkeley, California 94720, USA
[3]Department of Mechanical Engineering, University of Colorado Boulder, Colorado 80309 USA
[4]Department of Electrical and Computer Engineering, National University of Singapore, 4 Engineering Drive 3, Singapore 117583, Republic of Singapore
[5]Department of Physics and Centre for Computational Science and Engineering, National University of Singapore, Singapore 117546, Republic of Singapore
[6]NUS Graduate School for Integrative Sciences and Engineering, National University of Singapore, Kent Ridge 119620, Republic of Singapore
[7]NSF Nanoscale Science and Engineering Centre, 3112 Etcheverry Hall, University of California, Berkeley, California 94720, USA
[8]Materials Sciences Division, Lawrence Berkeley National Laboratory, 1 Cyclotron Road, Berkeley, California 94720, USA

SCATTERING SOLUTION TO THE HEAT EQUATION

Given the heat equation with homogeneous materials

$$\rho c_p \partial_t T = \nabla \cdot (\kappa \nabla T) \tag{1}$$

in polar coordinates we take the Fourier transform of time and use a separable solution $T(r,\theta,t) = R(r)e^{il\theta}e^{i\omega t}$ giving

$$\frac{i\omega\rho_0 c_{p0}}{\kappa_0} R = \frac{1}{r}\frac{d}{dr}(rR') - \frac{l^2}{r^2}R. \tag{2}$$

This is the differential equation for a modified Bessel function ($I_l(z)$ or $K_l(z)$) of $z = \sqrt{\frac{i\omega\rho_0 c_{p0}}{\kappa_0}}r$ for $\omega \neq 0$. The time-dependent solution is therefore

$$T_l^{(tr)}(r,\theta,\omega) = \left(a_l I_l(z) + b_l K_l(z)\right) e^{il\theta + i\omega t} \tag{3}$$

For the steady state of $\omega = 0$ the solutions become the solution to Laplace's equation

$$T_l^{(SS)}(r,\theta) = \left(A_l r^l + B_l r^{-l}\right) e^{il\theta} \tag{4}$$

for $l \neq 0$ and

$$T_0^{(SS)} = A_0 + B_0 \ln(r) \tag{5}$$

for $l = 0$. The general solution is therefore $T(r,\theta,\omega) = \Sigma_{l=0}^{\infty} T_l^{(SS)} + T_l^{(tr)}$.

For a perfect cloak

$$\kappa_r = \kappa_0 \frac{r-a}{r}, \quad \kappa_\theta = \kappa_0 \frac{r}{r-a},$$
$$\rho c_p = \rho_0 c_{p0} \left(\frac{b}{b-a}\right)^2 \frac{r-a}{r} \tag{6}$$

we can make the coordinate transformation

$$r' = \frac{b}{b-a}(r-a) \tag{7}$$

to reduce the solution in the primed coordinates to the homogeneous case.

---

* e-mail: Baowen.Li@Colorado.EDU

For a steady-state cloak (κ as for the perfect cloak, $\rho c_p = \rho_0 c_{p0}(b/(b-a))\eta$, i.e. evaluating ρc_p at $r = b$ when $\eta = 1$) no transformation will reproduce a homogeneous solution. Using $x = \sqrt{i\omega\rho_0 c_{p0}\eta b/\kappa_0(b-a)}(r-a)$ and separation of variables we find

$$0 = \partial_x(x\partial_x R) - \left[\frac{l^2}{x} + x + Ka\right] R \tag{8}$$

where $K = \sqrt{i\omega\rho_0 c_{p0}\eta b/\kappa_0(b-a)}$. This can be solved by the method of Frobenius $R_l(x) = \Sigma b^{\pm}_{nl} x^{n\pm l}$ with recurrence relation

$$b^{\pm}_{nl} = \frac{1}{n(n \pm 2l)}(Kab^{\pm}_{n-1,l} + b^{\pm}_{n-2,l}). \tag{9}$$

This relation is exact, but additional insight can be gained by expanding the solution by powers of Ka. For even terms in the series this is

$$b^{\pm(0)}_{2m,l} = \frac{1}{2m(2m \pm 2l)} b^{\pm(0)}_{2m-2,l} + O\left([Ka]^2\right) \tag{10}$$

which is the same as series expansion for I_l and K_l respectively. On the other hand, for odd terms it becomes

$$b^{\pm(0)}_{2m+1,l} = Ka \sum_{n=0}^{m} \frac{|2n-1|!!}{(2m+1)!!} \frac{(2n \pm 2l - 1)!!}{(2m \pm 2l + 1)!!} b^{\pm(0)}_{2n,l} + O\left([Ka]^3\right) \tag{11}$$

Because $b^{\pm(0)}_{2m+1,l}$ is completely determined by $b^{\pm(0)}_{2n,l}$ the odd terms are therefore a function of the modified Bessel functions. Ergo, we term these components $\mathcal{F}[R_l(x)]$. A similar derivation can be carried out for a spherical cloak where l becomes half-integer instead of integer.

SIMULATIONS OF THE SSC

Simulation Details

We model a rectangular domain of dimensions L =70 mm by $L_\perp$ =50 mm centered around a cloak of dimension a =13 mm, b =20 mm. The background medium is $\kappa_0 = 71.4\text{W/m}\cdot\text{K}$, $\rho_0 = 2100\text{kg/m}^3$, and $c_{p0} = 1000\text{J/kg}\cdot\text{K}$. This gives a diffusivity of $D = \kappa_0/\rho_0 c_{p0} = 3.4 \cdot 10^{-5}\text{m}^2/\text{s}$ and diffusion timescale $\tau_D = L^2/D = 144.12\text{s}$. The initial temperature was 293.15K with thermal baths at 300K, and T_0 =293.15K giving a ΔT of 6.85K. After confirming that the simulations were invariant under a change of scale we use the natural units of $x/L, y/L, t/\tau_D, (T - T_0)/\Delta T$.

Space Dependence of the Deviation of the SSC

In Fig S1 we take several slices of δT along y =constant for t = 2.08τ_D/100, 2.08τ_D/10, and 2.08τ_D (or 3s, 30s, and 300s) (blue, green, and red respectively) to observe the spatial dependence more precisely. Slices are centered, offset, and outside the cloak. Initially the perturbation is well confined to the portion of the cloak that has been reached by the applied heat current. As time passes and heat has spread relatively far into the domain the δT grows and spreads throughout the domain. As the system approaches steady state, δT falls. The linear dependence inside the cloak for steady state implies that $T^{(SSC)}$ inside this domain is essentially constant. Outside the cloak δT is effectively a sine curve. This is clearest for the slice outside the cloak (after the initial curve, which contains higher that decay faster than the fundamental mode), but even for the other two their linear drop-off away from the surface of the cloak corresponds to the linear section of a sine curve.

TIME-DEPENDENCE OF THE TEMPERATURE DIFFERENCE

Consider the heat equation for some arbitrary domain

$$\rho C \partial_t T = \nabla \cdot (\kappa \nabla T) \tag{12a}$$
$$T(\vec{r}, 0) = T_i \tag{12b}$$
$$T(\partial r, t) = T_{r_i} \tag{12c}$$

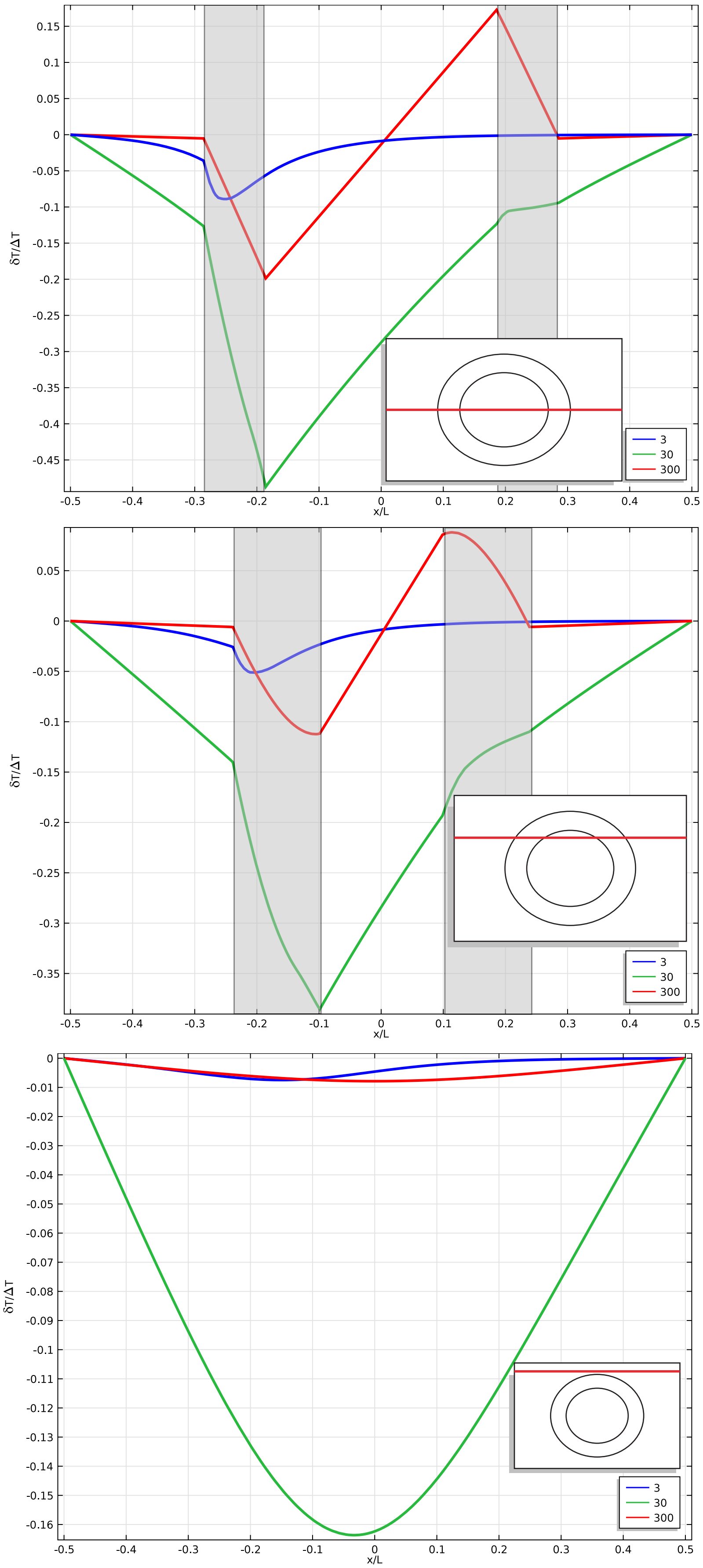

FIG. S1: Space dependence of δT of the SSC. Slices are along the middle of the cloak (plot (a), $y = 0$), slightly offset from the center (plot (b)$y = 1.1/7$), and outside the cloak (plot (c) $y = 2.2/7$). The blue, green, and red curves are at 3s ($2.08\tau_D/100$), 30s ($2.08\tau_D/10$), and 300s ($2.08\tau_D$) respectively.

where ∂r are the boundaries of the domain and the boundary conditions are stationary. In this case, there exists a steady state profile $\partial_t T^{(SS)} = 0$ that uniquely satisfies the boundary conditions. By linearity, $T = T^{(SS)} + T^{(tr)}$ where

$$\rho C \partial_t T^{(tr)} = \nabla \cdot \left(\kappa \nabla T^{(tr)} \right) \tag{13a}$$

$$T^{(tr)}(\vec{r}, 0) = T_i - T^{(SS)} \tag{13b}$$

$$T^{(tr)}(\partial r, t) = 0. \tag{13c}$$

Assuming that the materials are everywhere homogeneous for some coordinate system we can apply a spatial Fourier transform ($\nabla^2 T^{(tr)} \equiv -k^2 T^{(tr)}$) and therefore

$$T^{(tr)}(\vec{r}, t) = \int T^{(tr)}(\vec{k}, 0) e^{-k^2 D t} e^{-ik\cdot r} \frac{d^n k}{(2\pi)^{n/2}} \tag{14a}$$

$$T^{(tr)}(\vec{k}, 0) = \int e^{ik\cdot r} \left[T_i - T^{(SS)}(\vec{r}) \right] \frac{d^n r}{(2\pi)^{n/2}} \tag{14b}$$

where $D = \kappa_0 / \rho_0 c_{p0}$ is the thermal diffusivity. For two systems that differ only in ρc_p the difference between the two

$$\delta T(\vec{r}, t; \Delta D) = \int T^{(tr)}(\vec{k}, 0) \left(e^{-k^2 D_a t} - e^{-k^2 D_b t} \right) e^{-ik\cdot r} \frac{d^n k}{(2\pi)^{n/2}}. \tag{15}$$

Note the time dependence is a sum of the difference of exponentials. This implies that for short times δT is approximately linear while for long times it decays exponentially. In the case that only a single Fourier mode is excited δT is separable. This is also approximately true if a small number of well separated Fourier modes dominate $T^{(tr)}(\vec{k}, 0)$.

SENSITIVITY OF A CLOAK TO THE INNER BOUNDARY

Following [1] we consider a PC that has lost a section of the inner boundary of thickness δ. Defining the domains I, II, III to be external to the cloak, the cloak, and the interior the boundary conditions (continuity of T and $\hat{n} \cdot \kappa \nabla T$) are

$$a_l^{(I)} I_l(\sqrt{i} k_B b) + b_l^{(I)} K_l(\sqrt{i} k_B b) = a_l^{(II)} I_l(\sqrt{i} k_C [b-a]) + b_l^{(II)} K_l(\sqrt{i} k_C [b-a]) \tag{16a}$$

$$\kappa_0 k_B \left[a_l^{(I)} I_l'(\sqrt{i} k_B b) + b_l^{(I)} K_l'(\sqrt{i} k_B b) \right] = \kappa_r k_C \left[a_l^{(II)} I_l'(\sqrt{i} k_C [b-a]) + b_l^{(II)} K_l'(\sqrt{i} k_C [b-a]) \right] \tag{16b}$$

$$a_l^{(III)} I_l(\sqrt{i} k_B [a+\delta]) = a_l^{(II)} I_l(\sqrt{i} k_C \delta) + b_l^{(II)} K_l(\sqrt{i} k_C \delta) \tag{16c}$$

$$\kappa_0 k_B a_l^{(III)} I_l'(\sqrt{i} k_B [a+\delta]) = \kappa_r k_C \left[a_l^{(II)} I_l'(\sqrt{i} k_C \delta) + b_l^{(II)} K_l'(\sqrt{i} k_C \delta) \right] \tag{16d}$$

where $k_B = \sqrt{\omega \rho_0 c_{p0} / \kappa_0}$ and $(b-a) k_C = b k_B$, $a_l^{(I)}$ is the incident field component, $b_l^{(I)}$ is the scattered component, $a_l^{(III)}$ is the penetrating field, and we have expanded our solution using the eigenfunctions found in Sec. 1 ($b_l^{(III)}$ is tautologically 0 since $K_l(0)$ diverges). Using these definitions of k and κ the first conditions become

$$a_l^{(I)} I_l(\sqrt{i} k_B b) + b_l^{(I)} K_l(\sqrt{i} k_B b) = a_l^{(II)} I_l(\sqrt{i} k_B b) + b_l^{(II)} K_l(\sqrt{i} k_B b) \tag{17a}$$

$$a_l^{(I)} I_l'(\sqrt{i} k_B b) + b_l^{(I)} K_l'(\sqrt{i} k_B b) = a_l^{(II)} I_l'(\sqrt{i} k_B b) + b_l^{(II)} K_l'(\sqrt{i} k_B b), \tag{17b}$$

which, given an arbitrary b implies that $a_l^{(II)} = a_l^{(I)}$ and $b_l^{(II)} = b_l^{(I)}$. Using the last two boundary conditions and the fact that the Wronskian $\mathcal{W}[I_l(z), K_l(z)] = -1/z$ [3] gives

$$a_l^{(III)} = \frac{-(\sqrt{i} k_B a)^{-1}}{\frac{\delta}{a+\delta} \frac{b}{b-a} K_l'(\sqrt{i} k_C \delta) I_l(\sqrt{i} k_B [a+\delta]) - I_l'(\sqrt{i} k_B [a+\delta]) K_l(\sqrt{i} k_C \delta)} a_l^{(I)} \tag{18a}$$

$$b_l^{(I)} = \frac{I_l(\sqrt{i} k_C \delta) I_l'(\sqrt{i} k_B [a+\delta]) - \frac{\delta}{a+\delta} \frac{b}{b-a} I_l'(\sqrt{i} k_C \delta) I_l(\sqrt{i} k_B [a+\delta])}{\frac{\delta}{a+\delta} \frac{b}{b-a} K_l'(\sqrt{i} k_C \delta) I_l(\sqrt{i} k_B [a+\delta]) - I_l'(\sqrt{i} k_B [a+\delta]) K_l(\sqrt{i} k_C \delta)} a_l^{(I)} \tag{18b}$$

which can be expanded in the limit $\delta \to 0$. For $l \neq 0$ this gives

$$a_l^{(III)} \approx \frac{(\frac{1}{2}\sqrt{i} k_C \delta)^l}{(l-1)!} \frac{(\sqrt{i} k_B a)^{-1}}{l(\frac{1}{2}\sqrt{i} k_B a)^{-1} I_l(\sqrt{i} k_B a) + \frac{1}{2} I_l'(\sqrt{i} k_B a)} a_l^{(I)} \tag{19a}$$

$$b_l^{(I)} \approx \frac{2(\frac{1}{2}\sqrt{i} k_C \delta)^{2l}}{l[(l-1)!]^2} \frac{l(\sqrt{i} k_B a)^{-1} I_l(\sqrt{i} k_B a) - I_l'(\sqrt{i} k_B a)}{4l(\sqrt{i} k_B a)^{-1} I_l(\sqrt{i} k_B a) + I_l'(\sqrt{i} k_B a)} a_l^{(I)}. \tag{19b}$$

which vanish at $\delta = 0$ For $l = 0$ this gives

$$a_0^{(III)} \approx -\frac{1}{(\sqrt{i}k_B a) I_0'(\sqrt{i}k_B a) \ln k_C \delta} a_0^{(I)} \tag{20a}$$

$$b_0^{(I)} \approx \frac{1}{\ln k_C \delta} a_0^{(I)} \tag{20b}$$

which also vanishes at $\delta = 0$ but converges more slowly than the previous case. For $\omega = 0$ repeating the same procedure gives

$$A_l^{(II)} = \left(\frac{b}{b-a}\right)^l A_l^{(I)} \tag{21a}$$

$$A_l^{(III)} = \frac{2b-2a}{2b-a}\left(\frac{b}{b-a}\frac{\delta}{a}\right)^l A_l^{(I)} \tag{21b}$$

$$B_l^{(I)} = \frac{-a}{2b-a}\left(\frac{b}{b-a}\delta\right)^{2l} A_l^{(I)} \tag{21c}$$

$$B_l^{(II)} = \frac{-a}{2b-a}\delta^{2l} A_l^{(I)} \tag{21d}$$

for $l \neq 0$ and $A_0^{(I)} = A_0^{(II)} = A_0^{(III)}$, $B_0^{(I)} = B_0^{(II)} = 0$ for $l = 0$. Thus for a PC ($\delta \to 0$) the temperature inside is a constant and the scattering field vanishes. This confirms that a PC is truly perfect, as expected.

SIMULATIONS AND EXPERIMENTAL STUDY OF THE BC

We follow [2] to model the BC as rectangular domain of dimensions L =45 mm by $L_\perp$ =45 mm centered around a cloak with hidden region of size a =6 mm, first layer of r_2 =9.5 mm, and second layer of b =12 mm. The background medium is $\kappa_0 = 2.3\mathrm{W/m \cdot K}$, $\rho_0 = 2000\mathrm{kg/m^3}$, and $c_{p0} = 1500\mathrm{J/kg \cdot K}$, the outer layer's medium is $\kappa_1 = 9.8\mathrm{W/m \cdot K}$, $\rho_1 = 8440\mathrm{kg/m^3}$, and $c_{p1} = 400\mathrm{J/kg \cdot K}$, the inner layer's medium is $\kappa_2 = 0.03\mathrm{W/m \cdot K}$, $\rho_2 = 50\mathrm{kg/m^3}$, and $c_{p2} = 1300\mathrm{J/kg \cdot K}$, and the interior medium is $\kappa_3 = 205\mathrm{W/m \cdot K}$, $\rho_3 = 2700\mathrm{kg/m^3}$, and $c_{p3} = 900\mathrm{J/kg \cdot K}$. This gives a diffusivity of $D_0 = \kappa_0/\rho_0 c_{p0} = 7.67 \cdot 10^{-7}\mathrm{m^2/s}$ and diffusion timescale $\tau_{D_0} = L^2/D = 2641.3$s. The initial temperature was 273.15K with thermal baths at 333.15K, and T_0 =273.15K giving a ΔT of 60K. For plotting we use the natural units of $x/L, y/L, t/\tau_{D_0}, (T - T_0)/\Delta T$. The results are shown in Fig. S2, confirming that the cloak is visible in the transient response

We also test the time-dependence of δT using Fig. S3. As expected, the time-dependence is a sum of exponential terms like those predicted in eq. 15. Because of the additional boundaries in this system we see that there are more Fourier modes excited. What's more, the addition of these Fourier modes implies that the solution is not fully separable. This is clear from the separation of δT at the nearest point around the cloak to the heat source. This can also be seen with the initial plot of δT in Fig. S2 where there is initially relative cooling outside the cloak that is not found elsewhere along it's surface.

To verify our simulations we follow the procedure of Fig. S2 with an experimental realization of the BC and its homogeneous background. Since the temperatures at each boundary are not perfectly fixed, we normalize the data using the infimum of T_0 =285.08K and supremum of T =325.96K giving ΔT =40.88K. Using the normalization $(T - T_0)/\Delta T$ shows good agreement with the theoretical result. Results are plotted in Fig. S4. There is a slight discrepancy in the temperature deviation between the simulations and experiment. This is due to a slight difference in temperature gradients applied to the BC and homogeneous cases. Hence, this effect is strongest at the boundaries of the system and more negligible near the cloak itself.

[1] Ruan, Z., Yan, M., Neff, C. W., & Qiu, M. Ideal Cylindrical Cloak: Perfect but Sensitive to Tiny Perturbations. *Phys. Rev. Lett.* **99**, 113903 (2007).

[2] Han, T., *et al.* Experimental Demonstration of a Bilayer Thermal Cloak. *Phys. Rev. Lett.* **112**, 054302 (2014).

[3] Abramowitz, M. & Stegun, I. A. *Handbook of Mathematical Functions* (National Bureau of Standards, Washington, D.C., 1972).

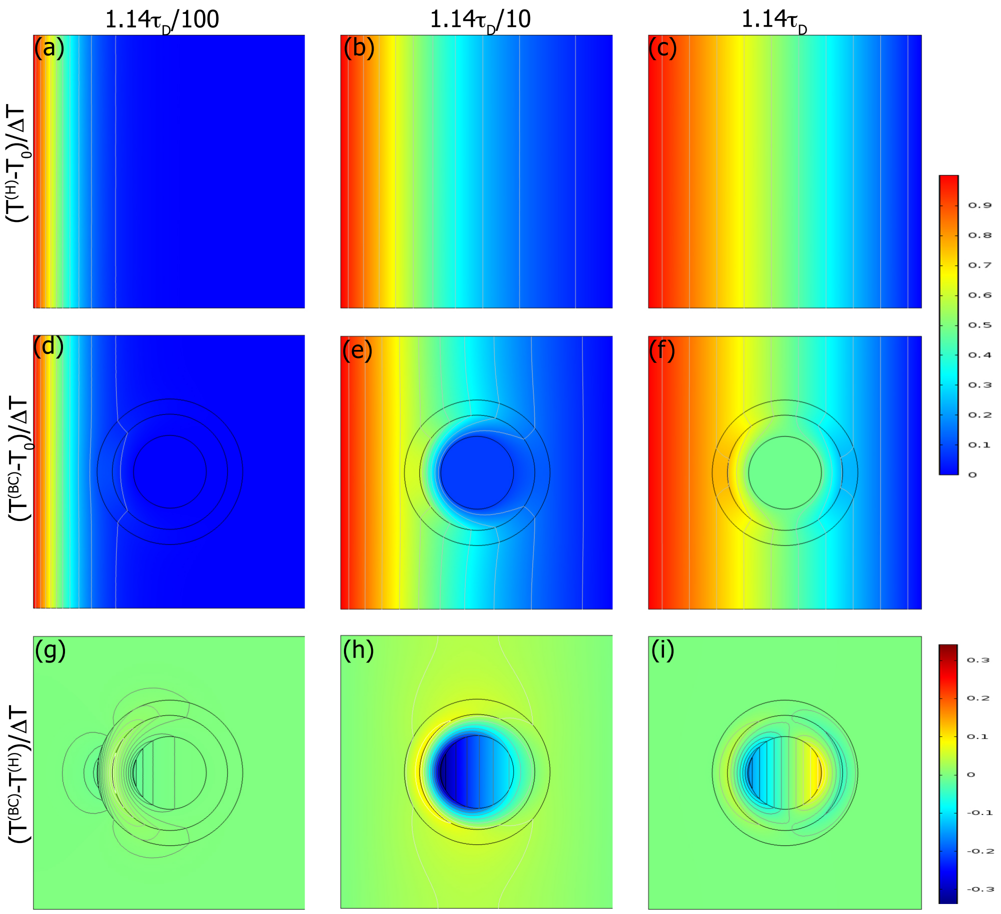

FIG. S2: Simulated temperature snapshots. Rows correspond to 30s ($1.14\tau_D/100$), 300s ($1.14\tau_D/10$), and 3000s ($1.14\tau_D$) respectively. Columns correspond to the homogeneous case (no cloak), BC, and $T^{(BC)} - T^{(H)}$. Black circles denote the location of the cloak (for reference in the homogeneous case), colored domains are isotherms, and grey lines are constant separation isotherms.

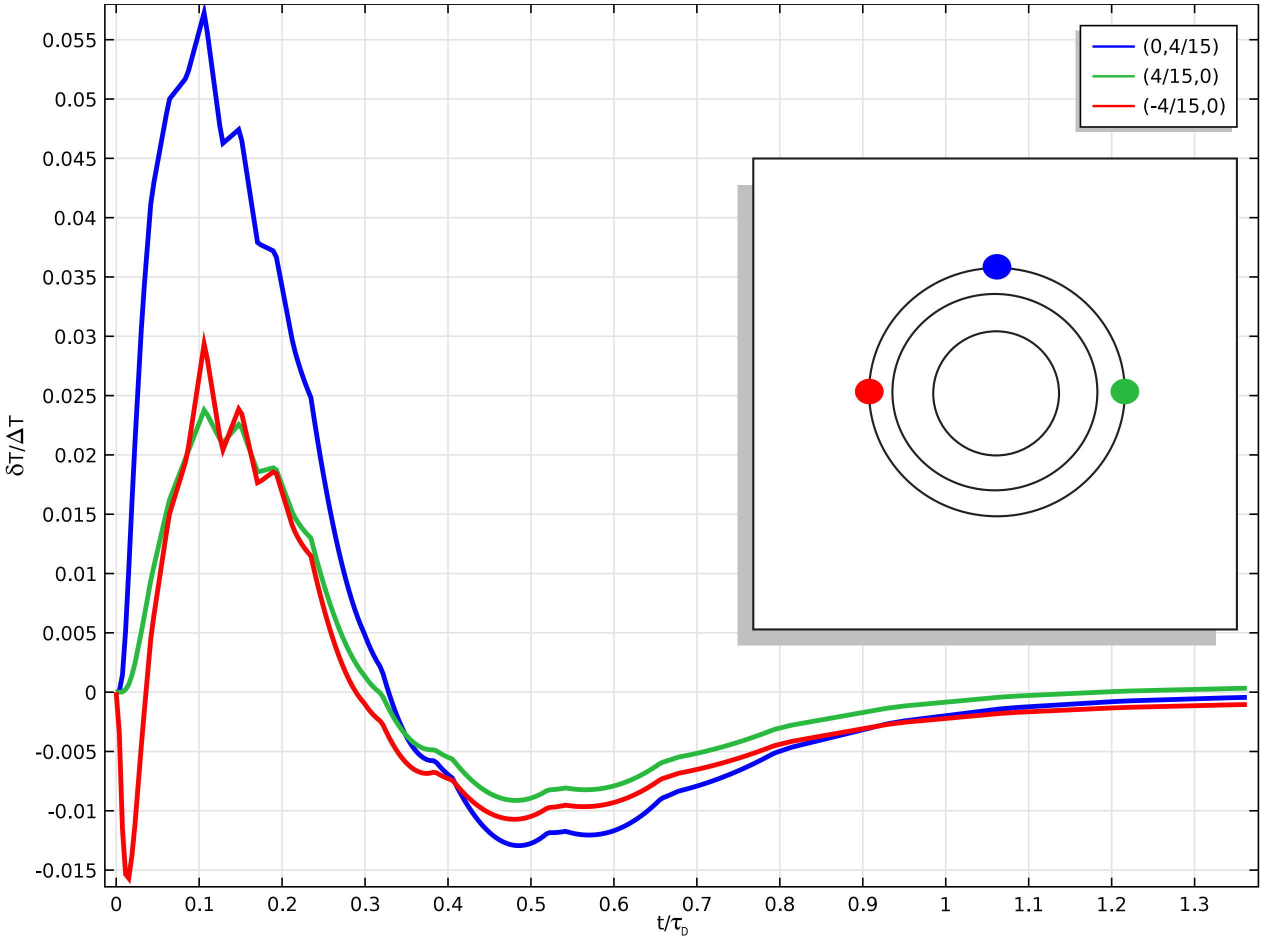

FIG. S3: Temperature deviation $\delta T/\Delta T$ for representative points outside the cloak as a function of time. Color corresponds to different points (see inset for key).

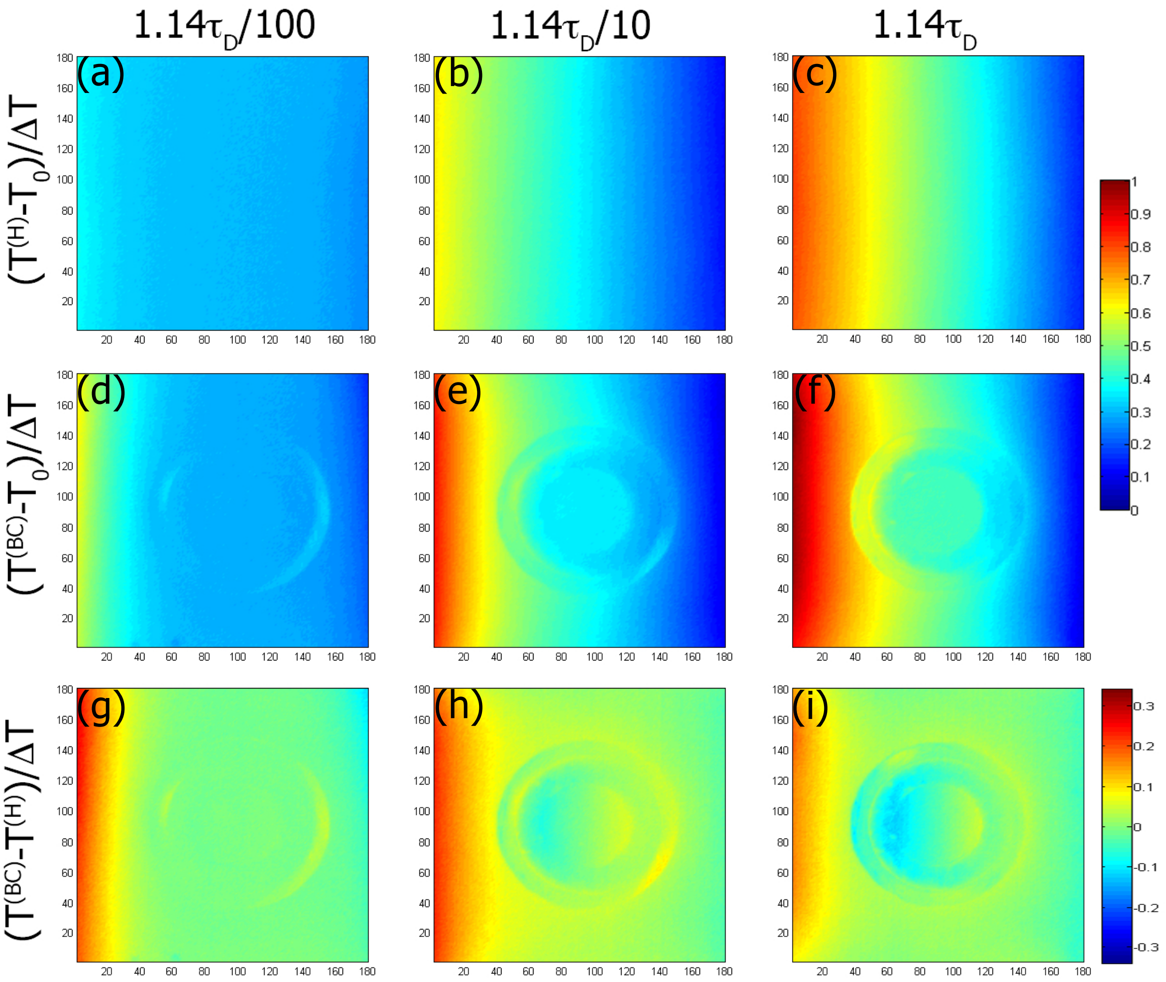

FIG. S4: Experimental temperature snapshots. Rows correspond to 30s ($1.14\tau_D/100$), 300s ($1.14\tau_D/10$), and 3000s ($1.14\tau_D$) respectively. Columns correspond to the homogeneous case (no cloak), BC, and $T^{(BC)} - T^{(H)}$. Black circles denote the location of the cloak (for reference in the homogeneous case), colored domains are isotherms, and grey lines are constant separation isotherms.
